# Self-reported awareness of the legal status of eight responsibilities of dog owners in Ireland: are dog owners different from non-dog owners?

**DOI:** 10.1186/s13620-021-00208-z

**Published:** 2022-01-05

**Authors:** Laura Keogh, Alison Hanlon, Andrew Kelly, Catherine Devitt, Locksley Messam

**Affiliations:** 1 School of Veterinary Medicine, University College Dublin, Dublin, Ireland; 2 The Irish Society for the Prevention of Cruelty to Animals, Longford, Ireland; 3 School of Biological Sciences, Queen’s University Belfast, Belfast, UK; 4 Independent Consultant, Glendalough, Wicklow Ireland

**Keywords:** Dog, Owner, Dog ownership, Law, Legal responsibility, Knowledge, Awareness, Canine welfare, Human health and wellbeing, One health, Prevalence

## Abstract

**Background:**

Legislation pertaining to canine ownership in Ireland maintains a one-health perspective by establishing a minimum standard of care for dogs while safeguarding human health and wellbeing. However, public awareness of this legislation has not been measured. The goals of this study were first, to estimate and compare the level of awareness, among dog owners and non-dog owners, that eight responsibilities of dog owners are prescribed by law in Ireland. Second, to determine if gender modifies differences in awareness between owners and non-owners, and third to determine whether gender itself is independently associated with awareness of the legal specification of these dog ownership responsibilities.

**Results:**

We conducted a cross-sectional study of 679 University College Dublin employees. Exposure information included participants’ dog ownership status, gender, age, and education level. Among dog owners and non-dog owners, we estimated and compared the prevalences of persons with self-reported awareness that each of eight dog ownership responsibilities are prescribed by law in Ireland: Dog fouling in a public place, the leashing and muzzling of certain breeds, holding a dog license, straying of dogs, safeguarding health and welfare of dogs, dog abandonment, prohibition on tail docking of puppies and the mandatory wearing of identification. The prevalence of awareness was low among both dog owners and non-dog owners with substantial awareness (≥ 80%) of only three responsibilities: Those pertaining to fouling, licensing and muzzling and leashing. Awareness that more than one responsibility was specified by law was also poor with only 17.9% (95% CI: 15.1–20.9%) of participants aware of all eight and dog owners essentially just as likely (54%; 95% CI: 49–58%) to be aware of more than one as non-dog owners. For most dog ownership responsibilities, differences in prevalence (PD) of awareness between owners and non-owners and females and males were trivial (PD < 10%). Similarly for most responsibilities, gender did not modify awareness PDs between owners and non-owners.

**Conclusions:**

In this well-educated university community, self-reported awareness that these eight responsibilities of dog owners are prescribed by law in Ireland is poor with essentially no difference between dog owners and non-dog owners or males and females. Awareness was higher for those responsibilities which, when not discharged, result in direct negative consequences to humans compared to those that result in direct negative consequences to dogs. It is likely that awareness of the legal status of these eight responsibilities of dog owners among the general public in Ireland is even less than observed in this study.

## Background

A recent report from eight European Union (EU) countries suggests that many young EU citizens believe more animal welfare legislation is needed [1]. This is consistent with findings from the Special Eurobarometer 442 report on animal welfare [2], in which citizens were found to have favourable attitudes towards companion animal welfare with 74% believing that improvement is needed. Eighty four percent of Irish respondents, in this latter study, thought that the welfare of companion animals in Ireland should receive stronger legal protections [2].

In Ireland, The Control of Dogs Act 1986 [3] and the Animal Health and Welfare Act 2013 [4] are the two main pieces of legislation governing the responsibilities of dog ownership. Maintaining a One Health-One Welfare perspective, these regulations prescribe a minimum standard of care for dogs, covering areas related to both animal welfare and human-animal interactions. This includes the duty of owners to protect animal welfare, the prohibition of animal abandonment and cruelty, the regulation of particular surgical procedures [4], as well as the requirement of dog licensing, the prohibition of dog straying and dog bye-laws [3]. These acts also safeguard human health and wellbeing, prescribing that dogs should be managed in a manner that does not cause community disruption and public harm. Currently, the extent to which the Irish public is aware of these acts is unknown and there are no studies reporting on the public’s knowledge of the legal status of any responsibilities of dog owners in Ireland. In the peer-reviewed literature, we have located only two studies [5, 6] investigating knowledge of laws or regulations governing treatment of owned dogs.

Societal knowledge of canine welfare-related legislation helps create a normative expectation for acceptable treatment of dogs, holding owners and non-owners to a high standard. Given that any law can only serve an ex ante function of guiding behaviour if it is known [7], it is important that all societal members (both dog owners and non-dog owners) be aware of the legal status of dog ownership responsibilities. Additionally, when issues of canine behaviour arise, the public will know what conduct is required of them, what protections are afforded to them by law and how they can act appropriately. Indeed, not only is legal knowledge a “first precondition” for compliance with the law [8], but its absence results in ignorance of the law’s benefits [9].

The goals of this study were: First, to estimate, among a) dog owners and b) non-dog owners, the prevalences of persons with awareness (hereafter just prevalence of awareness) that each of eight dog ownership responsibilities are prescribed by law in Ireland. Second, for each responsibility, to estimate differences in prevalence of awareness between owners and non-owners. Third, to determine if gender modifies differences in prevalence of awareness among dog owners and non-owners, and fourth to determine whether gender itself is independently associated with the prevalence of awareness. Consistent with the Cambridge English Dictionary usage, we define “awareness” as “knowledge that something exists” [10].

## Methods

### Study type and population

This study was part of a larger cross-sectional study on responsible dog ownership in Ireland [11] performed in collaboration with the Irish Society for the Prevention of Cruelty to Animals (ISPCA). The study population consisted of employees of University College Dublin (UCD), Ireland and exemption from ethical review was granted by UCD’s Human Research Ethics Committee (Reference Number: LS-E-15-78-Hanlon).

### Data collection

Data were collected between 24th^.^ August and 12th^.^ September 2015, during which all 3170 UCD employees were emailed a link to an online self-administered questionnaire via UCD’s communication office. Exposure information included participants’ age, gender, dog ownership status and education level. Outcome information collected included participants’ awareness that each of eight responsibilities of dog ownership are prescribed by law in Ireland. These responsibilities pertained to dog fouling in a public place [12], the leashing and muzzling of certain breeds [13], holding a dog license, straying of dogs [3], safeguarding health and welfare of dogs, dog abandonment, prohibition on tail docking of puppies and the mandatory wearing of identification [4] (hereafter referred to as “Fouling”, “Leashing and Muzzling”, “Licensing”, “Straying”, “Safeguarding Health and Welfare”, “Abandonment”, “Tail Docking”, and “Identification”, respectively). With regard to each of the eight dog ownership responsibilities, participants were asked if they believed there were laws governing them in Ireland (Table 1). Possible responses were: “Yes”, “No” or “I Don’t Know”.

**Table 1:** Outcome-related survey questions

**Are there laws in Ireland that say:**	
Owners can be fined if their dog fouls in a public place?	
All dog owners must have a license for their dog(s)?	
Certain breeds must be muzzled and on a leash in a public place?	
All owners must keep their animal in a way that safeguards its health and welfare?	
It is unlawful for an owner to abandon their dog?	
It is illegal to dock a puppy’s tail by anybody other than a veterinary surgeon?	
It is unlawful for an owner to allow their dog to stray?	
Dogs must at all times wear a collar that bears the name and address of the owner inscribed on a plate, badge or disc?	

### Statistical analysis

Of seven hundred and eleven UCD employees answering the question on their current dog-ownership status, 32 (4.5%) did not provide information on gender and were excluded from final data analysis. Data were analysed using SPSS software version 24 and Microsoft Excel version 16.25. All “I Don’t Know” and “No” responses were pooled and together considered evidence of lack of awareness that the statements are prescribed by legislation.

First, for both dog owners and non-dog owners (hereafter referred to as “owners” and “non-owners”, respectively), we estimated the prevalence of awareness that each of the eight dog-ownership responsibilities was prescribed by law (P_I_), as well as the owner versus non-owner differences in awareness prevalence (PD) for each one. Next, we estimated the prevalence of awareness that a certain number (zero to eight) of dog ownership responsibilities were prescribed by law (P_M_). Finally, using the Mann-Whitney procedure [14], we estimated the probability (Prob_MW_) that a randomly chosen owner would be more aware that the dog ownership responsibilities were prescribed by law than a non-owner. Here, we defined greater awareness by a higher number of affirmative answers to the eight questions. For each parameter estimated, we calculated 95% confidence intervals (CIs) using the normal approximation, and exact methods when sample sizes were small [15]. In order to check for any modification by gender of the relationship between dog ownership and awareness, we repeated the above-mentioned procedures for females and males separately.

Finally, we compared the prevalence of awareness among females, to the prevalence of awareness among males regardless of dog ownership status by estimating P_I_, P_M_, PD and Prob_MW_ as above, along with their respective 95% CIs.

We employed a magnitude-based approach to inferences [16] and assumed a prevalence of awareness of at least 80%, ie eight or more persons out of ten (P_I_ ≥ 80%) to be evidence of adequate societal awareness that a given dog ownership responsibility was prescribed by law. Thus, for any given responsibility, if all or most of the values within the 95% CI were 80% or more, awareness was considered adequate or likely adequate, respectively. Otherwise, it was not. For PDs, we assumed that a 10% (i.e., ten persons per 100) or greater difference in prevalence of awareness between groups (owners and non-owners, females and males) would be considered substantial and of practical importance.

Thus

- If all values within the 95% CI fell within the region PD ≥ 10% or its inverse (PD ≤ − 10%), we considered the difference substantial and of practical importance.
- If most of the values within the 95% CI fell within the region PD ≥ 10% or its inverse (PD ≤ − 10%), we considered the difference to be likely substantial and of likely practical importance.
- If all of the values within the 95% CI lay in the region − 10% < PD < 10%, we considered the difference to be trivial and of no practical importance.
- If most of the values within the 95% CI lay in the region − 10% < PD < 10%, we considered the difference to be likely trivial and likely of no practical importance.

When estimating the chance that an owner would be more aware that the dog ownership responsibilities were prescribed by law than a non-owner, we assumed that Prob_MW_ ≥ 60% (or Prob_MW_ ≤ 40%), would be substantial evidence of greater (or less) awareness. This range was used for inferences in an analogous manner to that used above for PDs.

## Results

### Study population characteristics

The six hundred and seventy-nine responses used for final data analysis represented a response rate of 21% and included 238 (35.1%) males and 441 (64.9%) females. There were slightly fewer owners (327) than non-owners (352) and a higher percentage of females among both owners (66.1%) and non-owners (63.8%). The ranges of the median age (41–45 years), lower (36–40 years) and upper (51–55) quartiles were the same for both owners and non-owners. Almost all owners (92.7, 95% CI: 89.8–95.5%) and non-owners (96.3, 95% CI: 94.3–98.3%) were educated at tertiary level.

### Awareness that individual dog ownership responsibilities are prescribed by law: owners versus (vs.) non-owners

**Fig. 1:**
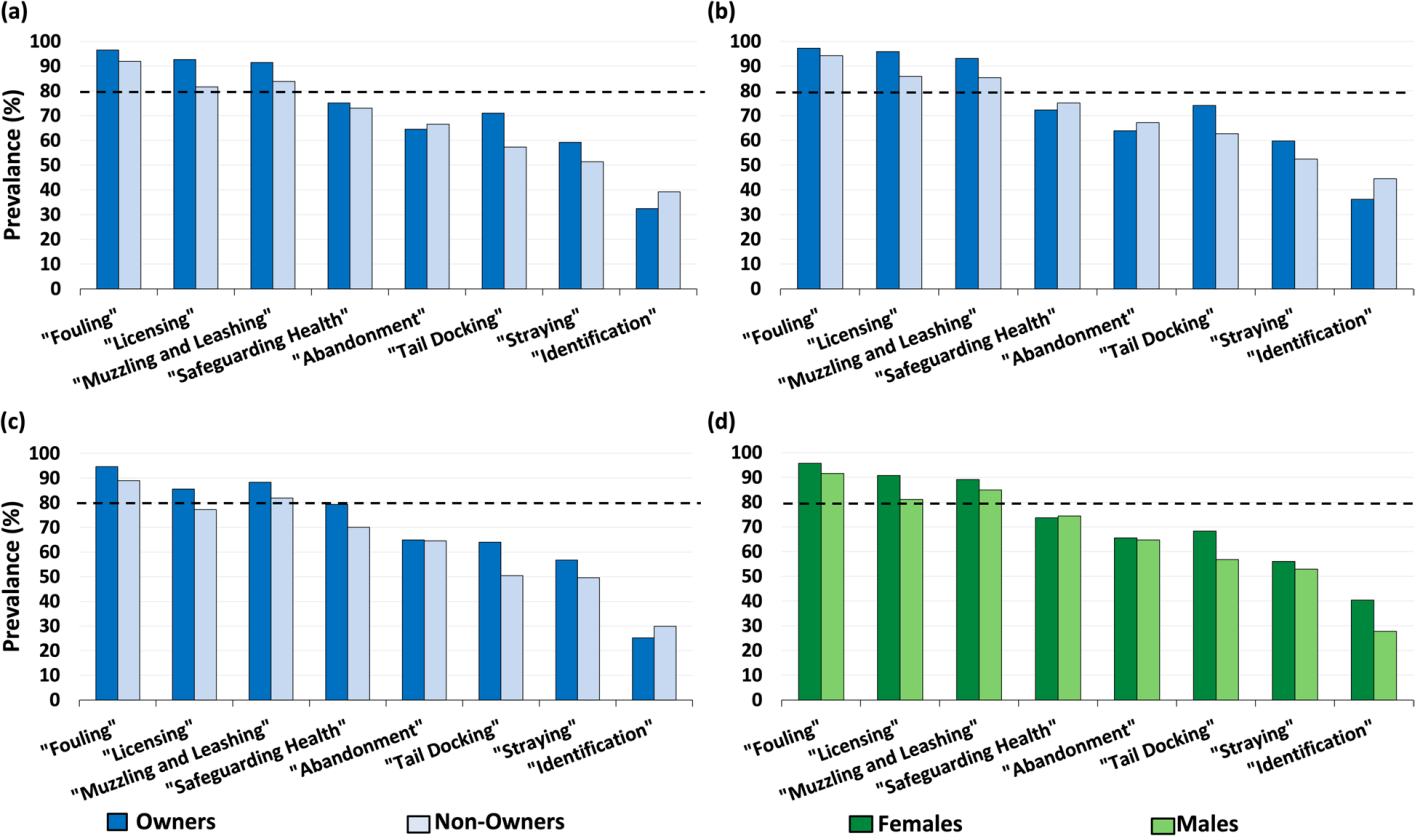
(**a**) Overall: Owners (327) vs. Non-Owners (352). (**b**) Female: Owners (216) vs. Non-Owners (225). (**c**) Male: Owners (111) vs. Non-Owners (127). (**d**) Females (441) and Males (238). Bar graphs of prevalence of University College Dublin employees with awareness of the legal status of eight responsibilities of dog owners in Ireland

Overall, the prevalence of awareness that the dog ownership responsibilities were prescribed by law was low. Among both owners and non-owners, there was adequate awareness (P_I_ ≥ 80%) with regard to only three of the eight responsibilities (“Fouling”, “Licensing” and “Muzzling and Leashing”) (Fig. 1a and Table 2). Awareness with regard to “Fouling” (P_I_ = 94.3, 95% CI: 92.5–96.0%), and “Identification” (P_I_ = 35.9, 95% CI: 32.3–39.5%) were highest and lowest, respectively (Table 2). In stratified analyses, a similar pattern was seen for both female (Fig. 1b and Table 2) and male (Fig. 1c and Table 2) owners and non-owners. Exceptions were the awareness with regard to “Abandonment” for which the prevalence was similar to “Tail Docking” among females but higher among males (Table 2).

**Table 2:** Gender-dog ownership status specific, and overall prevalence (P_I_) and 95% confidence intervals (CI) of University College Dublin employees with awareness that individual responsibilities of dog owners are prescribed by law in Ireland

Dog owner legal responsibility	Participant characteristics		Awareness of dog owner legal responsibility		P_**I**_ (%)	95% CI (%)
			Yes (n)	No (n)		
“Fouling”	Female	Owner	210	6	97.2	95.0–99.4
		Non-Owner	212	13	94.2	91.2–97.3
	Male	Owner	105	6	94.6	90.4–98.8
		Non-Owner	113	14	89.0	83.5–94.4
		Total	640	39	94.3	92.5–96.0
“Licensing”	Female	Owner	207	9	95.8	93.2–98.5
		Non-Owner	193	32	85.8	81.2–90.3
	Male	Owner	95	16	85.6	79.1–92.1
		Non-Owner	98	29	77.2	69.9–84.5
		Total	593	86	87.3	84.8–89.8
“Muzzling and Leashing”	Female	Owner	201	15	93.1	89.7–96.5
		Non-Owner	192	33	85.3	80.7–90.0
	Male	Owner	98	13	88.3	82.3–94.3
		Non-Owner	104	23	81.9	75.2–88.6
		Total	595	84	87.6	85.2–90.1
“Safeguarding Health and Welfare”	Female	Owner	156	60	72.2	66.3–78.2
		Non-Owner	169	56	75.1	69.5–80.8
	Male	Owner	88	23	79.3	71.7–86.8
		Non-Owner	89	38	70.1	62.1–78.0
		Total	502	177	73.9	70.6–77.2
“Abandonment”	Female	Owner	138	78	63.9	57.5–70.3
		Non-Owner	151	74	67.1	61.0–73.3
	Male	Owner	72	39	64.9	56.0–73.8
		Non-Owner	82	45	64.6	56.3–72.9
		Total	443	236	65.2	61.7–68.8
“Tail Docking”	Female	Owner	160	56	74.1	68.2–79.9
		Non-Owner	141	84	62.7	56.4–69.0
	Male	Owner	71	40	64.0	55.0–72.9
		Non-Owner	64	63	50.4	41.7–59.1
		Total	436	243	64.2	60.6–67.8
“Straying”	Female	Owner	129	87	59.7	53.2–66.3
		Non-Owner	118	107	52.4	45.9–59.0
	Male	Owner	63	48	56.8	47.5–66.0
		Non-Owner	63	64	49.6	40.9–58.3
		Total	373	306	54.9	51.2–58.7
“Identification”	Female	Owner	78	138	36.1	29.7–42.5
		Non-Owner	100	125	44.4	38.0–50.9
	Male	Owner	28	83	25.2	17.2–33.3
		Non-Owner	38	89	29.9	22.0–37.9
		Total	244	435	35.9	32.3–39.5

“Fouling” – Fouling in a public place; “Licensing” - Licensing of all dogs; “Muzzling and Leashing” – Muzzling and leashing of particular breeds; “Safeguarding health and welfare” - Safeguarding health and welfare of the animal; "Abandonment" - Abandonment of the dog; “Tail Docking” – Tail docking of puppies; “Straying” – Straying of an animal; “Identification” – Wearing of identification

### Differences in awareness that individual dog ownership responsibilities are prescribed by law: owners vs. non-owners

**Fig. 2:**
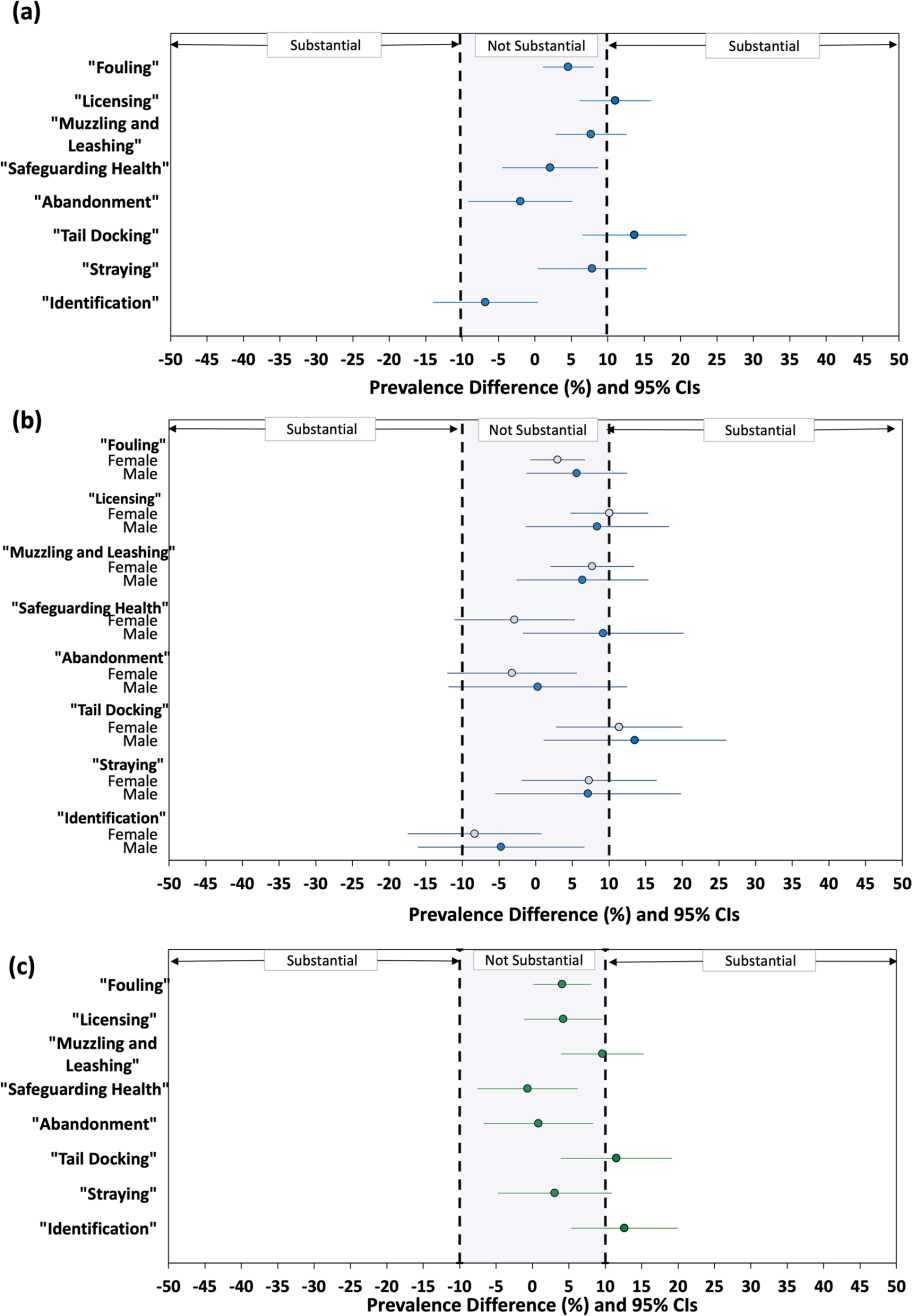
(**a**) Owners vs. Non-Owners. (**b**) Owners vs. Non-Owners: Stratified by gender. (**c**) Females vs. Males. Prevalence differences (%) and 95% confidence intervals (CI) for University College Dublin employees’ awareness of the legal status of eight responsibilities of dog owners in Ireland: Females compared to males

For only two responsibilities, “Licensing” (PD = 11.0%; 95% CI: 6.1–16.0%) and “Tail-Docking” (PD = 13.7%; 95% CI: 6.5–20.8%) were the owner vs. non-owner differences in awareness prevalence likely substantial, with both in favour of owners (Fig. 2 a).

When stratified on gender, results for owner vs. non-owner PDs were similar to those obtained in the unstratified analysis (Fig. 2a) except for “Safeguarding Health” which though inconsequential for both genders, had greater prevalence for non-owners among females (PD = − 2.9, 95% CI: − 11.1-5.3%) and for owners among males (PD = 9.2, 95% CI: − 1.8 - 20.2%) (Fig. 2b).

### Awareness that more than one dog ownership responsibility is prescribed by law: owners vs. non-owners

**Fig. 3:**
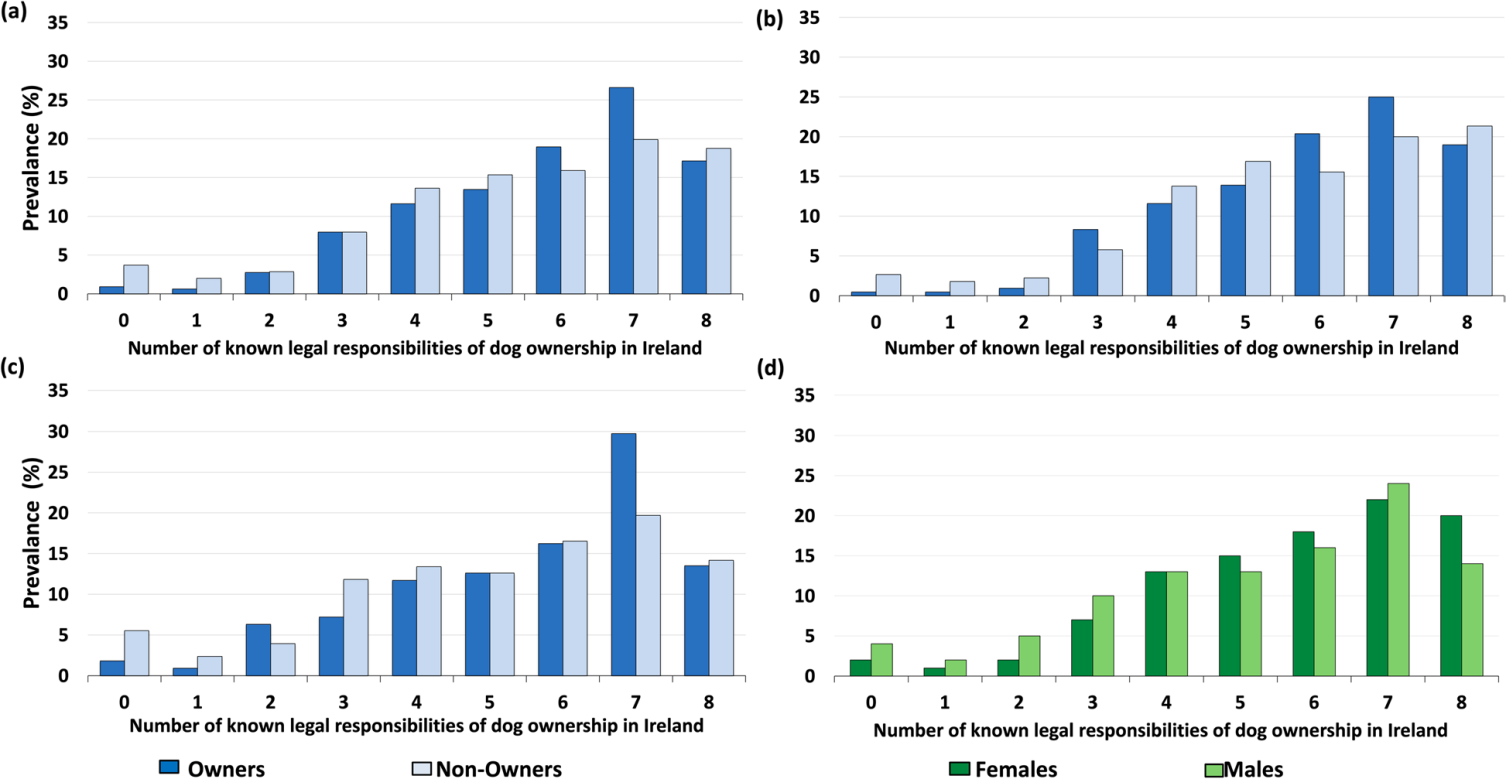
**a** Overall: Owners (327) vs. Non-Owners (352). **b** Female: Owners (216) vs. Non-Owners (225). **c** Males Owners (111) vs. Non-Owners (127). **d** Females (441) and Males (238). Bar graphs of prevalence of University College Dublin employees with awareness of the legal status of multiple (0 to 8) responsibilities of dog owners in Ireland

Among both owners and non-owners, the prevalence of awareness that more than one dog ownership responsibility is prescribed by law was low. Overall, 17.9% (95% CI: 15.1–20.9%) of participants (both owners and non-owners) were aware that all eight dog ownership responsibilities are prescribed by law, 76.1% (95% CI: 71.5–80.8%) of owners and 69.9% (95% CI: 64.9–74.9%) of non-owners were aware of five or more and 0.9% (95% CI: 0.2–2.6%) of owners and 3.7% (95% CI: 2.0–6.2%) of non-owners were not aware of any (Fig. 3a). There was a 54% (95% CI: 49–58%) chance that a randomly chosen owner would be more aware that the dog ownership responsibilities were prescribed by law than a non-owner.

When stratified by gender, the pattern of awareness of the legal status of more than one responsibility was similar to the overall pattern (Fig. 3b-c and Table 3). Seventy-eight percent (95% CI: 72.5–83.5%) of female owners, 74% (95% CI: 68.3–79.7%) of female non-owners, 72% (95% CI: 63.6–80.4%) of male owners and 63% (95% CI: 54.6–71.4%) of male non-owners were aware that five or more statements were prescribed by law. The chance that a randomly chosen owner would be more aware that the dog ownership responsibilities were prescribed by law than a non-owner was similar among females (Prob_MW_ = 52%; 95% CI: 47–58%) and males (Prob_MW_ = 56%; 95% CI: 49–62%) and not substantial.

**Table 3:** Gender-Dog ownership status specific, and overall prevalence (P_M_) and 95% confidence intervals (CI) of University College Dublin employees with awareness that multiple (zero to eight) responsibilities of dog owners are prescribed by law in Ireland

Number of dog owner legal responsibilities (M)	Participant characteristics		Awareness of exactly M legal responsibilities		P_**M**_ (%)	95% CI (%)
			Yes (n)	No (n)		
0	Female	Owner	1	215	0.5	0.01–2.6^a^
		Non-Owner	6	219	2.7	1.0–5.7^a^
	Male	Owner	2	109	1.8	0.2–6.4^a^
		Non-Owner	7	120	5.5	2.2–11.0^a^
		Total	16	663	2.4	1.4–3.8^a^
1	Female	Owner	1	215	0.5	0.01–2.6^a^
		Non-Owner	4	221	1.8	0.5–4.5^a^
	Male	Owner	1	110	0.9	0.02–4.9^a^
		Non-Owner	3	124	2.4	0.5–6.7^a^
		Total	9	670	1.3	0.6–2.5^a^
2	Female	Owner	2	214	0.9	0.1–3.3^a^
		Non-Owner	5	220	2.2	0.7–5.1^a^
	Male	Owner	7	104	6.3	2.6–12.6^a^
		Non-Owner	5	122	3.9	1.3–8.9^a^
		Total	19	660	2.8	1.7–4.3^a^
3	Female	Owner	18	198	8.3	5.0–12.9^a^
		Non-Owner	13	212	5.8	3.1–9.7^a^
	Male	Owner	8	103	7.2	3.2–13.7^a^
		Non-Owner	15	112	11.8	6.8–18.7^a^
		Total	54	625	7.9	6.0–10.2^a^
4	Female	Owner	25	191	11.6	7.6–16.6
		Non-Owner	31	194	13.8	9.6–19.0
	Male	Owner	13	98	11.7	6.4–19.2
		Non-Owner	17	110	13.4	8.0–20.6
		Total	86	593	12.7	10.3–15.4
5	Female	Owner	30	186	13.9	9.6–19.2
		Non-Owner	38	187	16.9	12.2–22.4
	Male	Owner	14	97	12.6	7.1–10.3
		Non-Owner	16	111	12.6	7.4–19.7
		Total	98	577	14.4	11.9–17.4
6	Female	Owner	44	172	20.4	15.2–26.4
		Non-Owner	35	190	15.6	11.1–21.0
	Male	Owner	18	93	16.2	9.9–24.4
		Non-Owner	21	106	16.5	10.5–24.2
		Total	118	561	17.4	14.6–20.4
7	Female	Owner	54	162	25.0	19.4–31.3
		Non-Owner	45	180	20.0	15.0–25.8
	Male	Owner	33	78	29.7	21.4–39.1
		Non-Owner	25	102	19.7	13.2–27.7
		Total	157	522	23.1	20.0–26.5
8	Female	Owner	41	175	18.9	14.0–24.9
		Non-Owner	48	177	21.3	16.2–27.3
	Male	Owner	15	96	13.5	7.8–21.3
		Non-Owner	18	109	14.2	8.6–21.5
		Total	122	557	17.9	15.1–20.9

^a^95% Confidence Intervals calculated using exact methods

### Awareness that individual dog ownership responsibilities are prescribed by law: females vs. males

Similar to the overall pattern, among both females and males (regardless of ownership status) there was adequate (i.e., P_I_ > 80%) awareness regarding only “Fouling”, “Licensing”, and “Muzzling and Leashing” (Fig. 1d and Table 4) with greatest and least awareness pertaining to “Fouling” and “Identification”, respectively, among both genders (Fig. 1d and Table 4).

**Table 4:** Gender- specific and overall prevalence (P_I_) and 95% confidence intervals (CI) of University College Dublin employees’ awareness that individual responsibilities of dog owners are prescribed by law in Ireland

Dog owner legal responsibility	Participant characteristics	Awareness of dog owner legal responsibility		P_**I**_ (%)	95% CI (%)
		Yes (n)	No (n)		
“Fouling”	Female	422	19	95.7	93.8–97.6
	Male	218	20	91.6	88.1–95.1
	Total	640	39	94.3	92.5–96.0
“Licensing”	Female	400	41	90.7	88.0–93.4
	Male	193	45	81.1	76.1–86.1
	Total	593	86	87.3	84.8–89.8
“Muzzling and Leashing”	Female	393	48	89.1	86.2–92.0
	Male	202	36	84.9	80.3–89.4
	Total	595	84	87.6	85.2–90.1
“Safeguarding Health and Welfare”	Female	325	116	73.7	69.6–77.8
	Male	177	61	74.4	68.8–79.9
	Total	502	177	73.9	70.6–77.2
“Abandonment”	Female	289	152	65.5	61.1–69.9
	Male	154	84	64.7	58.6–70.8
	Total	443	236	65.2	61.7–68.8
“Tail Docking”	Female	301	140	68.3	63.9–72.6
	Male	135	103	56.7	50.4–63.0
	Total	436	243	64.2	60.0–67.8
“Straying”	Female	247	194	56.0	51.4–60.6
	Male	126	112	52.9	46.6–59.3
	Total	373	306	54.9	51.2–58.7
“Identification”	Female	178	263	40.4	35.8–44.9
	Male	66	172	27.7	22.0–33.4
	Total	244	435	35.9	32.3–39.5

“Fouling” – Fouling in a public place; “Licensing” - Licensing of all dogs; “Muzzling and Leashing” – Muzzling and leashing of particular breeds; “Safeguarding health and welfare” - Safeguarding health and welfare of the animal; "Abandonment" - Abandonment of the dog “Tail Docking” – Tail docking of puppies; “Straying” – Straying of an animal; “Identification” – Wearing of identification

### Differences in awareness that individual dog ownership responsibilities are prescribed by law: females vs. males

For each legal responsibility, the female-male difference in prevalence of awareness was in favour of females, though most were likely trivial (Fig. 2c). Only for “Identification” (PD = 12.6, 95% CI: 5.3–19.9%) and “Tail Docking” (PD =11.5, 95% CI: 3.9–19.2%) were observed differences likely substantial (Fig. 2c).

### Awareness that more than one dog ownership responsibility is prescribed by law: females vs. males

Among both females and males, awareness that more than one dog ownership responsibility is prescribed by law was low. Among females, 76.0% (95% CI: 72.0–80.0%) were aware of five or more and 20.2% (95% CI: 16.4–23.9%) were aware that all eight were prescribed by law (Table 5). Patterns were similar for males with 67.2% (95% CI: 62.8–71.6%) aware of five or more and 13.9% (95% CI: 9.5–18.3%) aware of all eight (Table 5). The chance that a randomly chosen female would be more aware that the dog ownership responsibilities were prescribed by law than a male was not substantial (Prob_MW_ = 56%, 95% CI: 53–58%).

**Table 5:** Gender specific and overall prevalence and 95% confidence intervals (CI) of University College Dublin employees’ awareness that multiple (zero to eight) responsibilities of dog owners are prescribed by law in Ireland

Number of dog owner legal responsibilities (M)	Participant characteristics	Awareness of exactly M legal responsibilities		P_**M**_ (%)	95% CI (%)
		Yes (n)	No (n)		
0	Female	7	434	1.6	0.6–3.2^a^
	Male	9	229	3.8	1.7–7.1^a^
	Total	16	663	2.4	1.4–3.8^a^
1	Female	5	436	1.1	0.4–2.6^a^
	Male	4	234	1.7	0.5–4.2^a^
	Total	9	670	1.3	0.6–2.5^a^
2	Female	7	434	1.6	0.6–3.2^a^
	Male	12	226	5.0	2.6–8.6^a^
	Total	19	660	2.8	1.7–4.3^a^
3	Female	31	410	7.0	4.8–9.8^a^
	Male	23	215	9.7	6.2–14.1^a^
	Total	54	625	8.0	6.0–10.2^a^
4	Female	56	385	12.7	9.7–16.2
	Male	30	208	12.6	8.7–17.5
	Total	86	593	12.7	10.3–15.4
5	Female	68	373	15.4	12.2–19.1
	Male	30	208	12.6	8.7–17.5
	Total	98	582	14.4	11.9–17.3
6	Female	79	362	17.9	14.4–21.8
	Male	39	199	16.4	11.9–21.7
	Total	118	561	17.4	14.6–20.4
7	Female	99	342	22.4	18.6–26.6
	Male	58	180	24.4	19.1–30.3
	Total	157	522	23.1	20.0–26.5
8	Female	89	352	20.2	16.5–24.2
	Male	33	205	13.9	9.7–18.9
	Total	122	557	18.0	15.2–21.1

^a^95% Confidence Intervals calculated using exact methods

## Discussion

Measuring knowledge or awareness of laws is considered problematic by some scholars [17, 18]. This difficulty stems in part, from the challenge in determining what component of any marker of knowledge or awareness results from a conscious recognition of a statute and what component is just an expression of an individual’s moral stance or level of consciousness of rights. Additionally, it has been argued that true knowledge of a law is determinable only in its specific situations [18] which are hard to exhaustively replicate in the context of research. In general, there have been three broad approaches used to measure public knowledge of laws [17]: Participant self-report [19–21], Elicitation of statements indicative of participants’ knowledge [20] and via categorical responses to questions about existing laws [7, 22–25]. This current study, along with others measuring knowledge of animal-welfare legislation [1, 5, 26], falls into both the first and the third categories. In this study we have measured awareness of laws with awareness defined as “knowledge that something exists” [10].

We have found that overall awareness that dog ownership responsibilities were prescribed by law is poor, regardless of dog ownership status or gender, and that there is substantial variation in awareness depending on the responsibility. This is consistent with previous findings in studies conducted on knowledge of the law in different jurisdictions and domains including animal welfare [1, 5, 26], medicine [22, 23, 25], education [27], employment [28, 29] and the family [30, 31]. The volume, complexity of legal jargon and format of statutes have previously been proffered as potential reasons for poor public legal knowledge [9, 17] and are likely to apply here as well. However, this does not explain the observed variation in awareness, with substantial awareness that some dog ownership responsibilities (“Fouling”, “Licensing” and “Muzzling and Leashing”) were prescribed by law (P_I_ ≥ 80%) while for others, for example “Identification” there was awareness by only a few (P_I_ < 40%) participants. We speculate that several factors might contribute to this variation. First, the immediate consequences of non-compliance with regulations governing “Fouling”, “Licensing” and “Muzzling and Leashing” are felt by humans (e.g., disgust at dog faeces in public places, fines levied against owners and dog bites to members of the public) and only through these effects on human society does non-compliance negatively affect canine welfare (prohibition of dogs in public spaces, confiscation and/or euthanasia). Conversely, non-compliance with regulations governing the rest of responsibilities (“Safeguarding Health and Welfare”, “Abandonment”, etc.), exert immediate and direct negative effects on the welfare of the dog in question. Thus, there may be greater public sensitivity to matters related to “Fouling”, “Licensing” and Muzzling and Leashing” than to the others. Consequently, rather than just an explicit knowledge of the law, this high prevalence of awareness may, in part, result from a strong correlation between the existence of the legislation and participants’ sense of what should be. Across different domains, it has been found that members of society often assume that the law corresponds to their own personal sense of what is correct [17]. If this is a strong determinant in the prevalence of awareness, it might also explain, in part, the small difference in observed awareness between owners and non-owners. Poor awareness that “Identification” is prescribed by law, could be in part a sign that persons do not see the necessity for this legislation. Another reason for the observed discrepancy in awareness might be that “Fouling” and “Licensing” are among the most well publicised laws in Dublin. The presence of dog waste bins, merchandising of dog pooh bags and holders and signs on buses, on the street and in parks likely contributes to increased awareness of “Fouling”. Dog licensing is frequently mentioned in print and electronic media due to the purported low rate of compliance among dog owners. Additionally, it was first introduced into law in Ireland in 1865 [32]. This might also contribute to awareness.

Our results also suggest that, notwithstanding a tendency towards greater awareness, dog-owners and females, display essentially the same levels of awareness as non-owners and males, respectively. Additionally, the relationship of dog ownership to awareness of the laws is not modified by gender. In previous studies, no clear pattern of relationship has emerged between demographic characteristics and legal knowledge [17], though at least one study of family law has found that women have better law knowledge than men [30]. This has been explained by suggesting that concern and therefore knowledge of the law is more likely among those that have greatest role responsibility in the domain. Contrary to Weng et al.’s findings [5], but consistent with Simonato et al. [6], our study does not provide support for this view with respect to dog ownership except in the case of “Tail Docking” and “Licensing”. Apart from high expected correlation between the social norms of owners and non-owners, potential reasons for trivial differences in awareness may include non-dog owners’ being previous dog owners as well as currently caring for and/or residing with dogs owned by others. We cannot explain why only in the case of “Tail Docking” and “Identification”, do females show substantially higher awareness than males.

These results suggest that there is need for public education aimed at increasing awareness of canine welfare laws. Targeted educational campaigns could serve to make the language of the law more palatable to the public. Additionally, the provision of educational material in local ISPCA offices and veterinary practices may help to increase awareness.

This study has a number of limitations. The response rate (21%) does not preclude non-response bias affecting our estimates and it is possible that UCD staff interested in canine welfare were more likely to participate than those who are not. This could have led to overestimates of prevalence of awareness. Second, while we think that a negative or “I Don’t Know” response is likely to be an accurate sign of a lack of awareness (close to perfect sensitivity) an affirmative response, is less of an indicator of conscious awareness (imperfect specificity) and could, in part, be an indication of correspondence of that law with the participant’s moral stance. This latter has been a common observation in knowledge of the law studies [17]. Third, transportability of prevalence estimates is affected by the high standard of education (> 90% at tertiary-level) of these participants relative to the general Irish population. Nevertheless, higher education has not been found to consistently predict higher awareness of laws [17], with Weng et al. finding an inverse relationship between an understanding of the law and educational levels [5]. Fourth, we did not have information on current or past relationships of non-dog owners with dogs. This information might have helped to explain the similar levels of observed awareness with owners. Finally, we have used a prevalence of at least 80% as a marker of substantial awareness. This value is subjective and other values, could reasonably be used. Nevertheless, it renders the bases of our inferences transparent.

Notwithstanding limitations, we believe this study contributes uniquely to an understanding of awareness of laws governing dog ownership among university employees in Ireland. Not only does it quantify levels of awareness, but alerts readers to the possibility that public ignorance of laws governing dog ownership may be higher than apparent, given the likely discrepancy between the educational level of the study population and the general public.

## Conclusions

We conclude with the following points: 1) Among employees in this university community, there is overall poor awareness among both dog owners and non-dog owners that common dog ownership responsibilities are prescribed by law in Ireland, 2) There is substantial variation in the level of awareness of the legal status of different dog ownership responsibilities. Higher levels of awareness were observed for those responsibilities which directly affect human well-being compared to those that directly affect canine welfare. 3) With few exceptions, among well-educated individuals, differences in levels of awareness between dog owners and non-dog owners are trivial and not modified by gender. 4) Gender itself shows only a trivial association with awareness. Given the high level of education of the individuals studied, we think it likely that awareness that the selected dog ownership responsibilities are prescribed by law, is substantially lower among the general public in Ireland.

## Data Availability

The datasets used and analysed during the current study are available from the corresponding author upon reasonable request.

## Abbreviations

UCD: University College Dublin
ISPCA: Irish Society for Prevention of Cruelty to Animals
